# Sorting nexin-dependent therapeutic targeting of oncogenic epidermal growth factor receptor

**DOI:** 10.1038/s41417-022-00541-7

**Published:** 2022-10-17

**Authors:** Benjamin Atwell, Cheng-Yu Chen, Matthew Christofferson, William R. Montfort, Joyce Schroeder

**Affiliations:** 1 Department of Molecular and Cellular Biology, 1007 E Lowell St, Tucson, AZ 85721 USA; 2 Department of Chemistry and Biochemistry, 1007 E Lowell St, Tucson, AZ 85721 USA; 3 University of Arizona Cancer Center, 1007 E Lowell St, Tucson, AZ 85721 USA; 4 BIO5 Institute, University of Arizona, 1007 E Lowell St, Tucson, AZ 85721 USA

**Keywords:** Breast cancer, Cell biology

## Abstract

Overexpression and/or overactivation of the Epidermal Growth Factor Receptor (EGFR) is oncogenic in several tumor types yet targeting the kinase domain of wildtype EGFR has had limited success. EGFR has numerous kinase-independent roles, one of which is accomplished through the Sorting Nexin-dependent retrotranslocation of EGFR to the nucleus, which is observed in some metastatic cancers and therapeutically resistant disease. Here, we have utilized the BAR domain of Sorting Nexin 1 to create a peptide-based therapeutic (cSNX1.3) that promotes cell death in EGFR-expressing cancer. We evaluated the efficacy of cSNX1.3 in tumor-bearing WAP-TGFα transgenic mice (an EGFR-dependent model of breast cancer), where cSNX1.3 treatment resulted in significant tumor regression without observable toxicity. Evaluation of remaining tumor tissues found evidence of increased PARP cleavage, suggesting apoptotic tumor cell death. To evaluate the mechanism of action for cSNX1.3, we found that cSNX1.3 binds the C-terminus of the EGFR kinase domain at an interface site opposite the ATP binding domain with a *K*_d_ of ~4.0 µM. In vitro analysis found that cSNX1.3 inhibits the nuclear localization of EGFR. To determine specificity, we evaluated cancer cell lines expressing wildtype EGFR (MDA-MB-468, BT20 and A549), mutant EGFR (H1975) and non-transformed lines (CHO and MCF10A). Only transformed lines expressing wildtype EGFR responded to cSNX1.3, while mutant EGFR and normal cells responded better to an EGFR kinase inhibitor. Phenotypically, cSNX1.3 inhibits EGF-, NRG-, and HGF-dependent migration, but not HA-dependent migration. Together, these data indicate that targeting retrotranslocation of EGFR may be a potent therapeutic for RTK-active cancer.

## Introduction

The HER family (including the Epidermal Growth Factor Receptor/HER1, HER2, HER3 and HER4) of tyrosine kinase receptors (RTKs) is highly prevalent within numerous cancer types (including breast, lung, colon, head and neck, among others), and, in some cases, antibody-based therapeutics (i.e. Trastuzumab) are highly effective [1]. Alternatively, in HER2 negative, but HER1 and HER3 positive breast cancer, antibody-based treatments have not shown efficacy [1]. In addition, while Tyrosine Kinase Inhibitors (TKIs) work well in many cancers, such as lung, head and neck and colon cancer, they have failed to be impactful in the breast [1]. This is true even in HER2-positive breast cancer, indicating alternative functions for the HER family in breast cancer. In fact, the HER receptors can function in a number of kinase-dependent and kinase-independent ways. For example, in metastatic and therapeutic-resistant breast cancer patient samples, EGFR/HER1 is not restricted to the cell surface, but instead can be found intracellularly in both endocytic organelles and the nucleus [2, 3]. Importantly, the kinase may be unnecessary to the function of nuclear EGFR, where the receptor functions as a transcriptional co-factor for the transcription of genes such as cyclinD1, iNOS and Aurora kinase [4]. The process by which EGFR is alternatively trafficked to the nucleus occurs for many RTKs and is known as retrotranslocation or retrograde trafficking [5]. During this process, RTKs are trafficked through a series of long-lived endosomes to the endoplasmic reticulum and nucleus [5].

This trafficking is regulated by multiple protein complexes, one of which involves a set of proteins called the Sorting Nexins (SNX) [6]. Of the multiple Sorting Nexin subgroups, one called the PX-BAR subgroup includes SNX1, which is the mammalian homologue of the yeast vacuolar protein Vsp5p. Vsp5p is an evolutionarily conserved protein that serves as a core component of the Retromer, a protein complex that regulates retrograde trafficking of transmembrane proteins [7]. The SNX proteins in the PX-BAR subgroup contain two key functional domains, a PX domain in their N-terminus that interacts with phosphatidylinositol (PI(3)P) moieties in the membrane, and a BAR domain composed of coiled-coiled alpha-helices that drive protein-protein interactions and can promote membrane remodeling [7]. The BAR domain also drives SNX homo- and hetero-dimerization, key events in sorting and trafficking. The dimerized BAR domains of a pair of sorting nexins then bind to cargo proteins that will be trafficked along tubulovesicles. SNX1 was originally identified as a protein that interacts with EGFR [8] and subsequent to this discovery, it was found that many sorting nexins can regulate the trafficking of EGFR, including SNX2 [9], SNX6 [10], SNX9 [11] and SNX16 [12]. Additional studies demonstrated that the chronic overexpression of SNX1 results in the formation of extensive tubular networks due to membrane bending by the BAR domain, and which could be leading to retrograde trafficking of cargo [13].

Of note, sorting nexins have now been shown to regulate the trafficking of additional RTKs, including c-Met [14] and IGF1R [15]. We hypothesize in the current study that a therapeutic that can modify the interaction between Sorting Nexins and RTKs may target tumor-specific retrotranslocation. This approach could have clinical impact due to the observation that tumors that develop TKI therapeutic resistance frequently overexpress an alternative RTK HER family in cancer progression’. To target these interactions, we utilized cell-penetrating peptides with a Protein Transduction Domain to allow for the delivery of peptides across the cell membrane [16]. Such cell-penetrating peptides have been used to block protein-protein interactions between a number of targets, including EGFR and MUC1 [17]. Here, we evaluated the impact of multiple SNX1 peptides in their ability to drive RTKs away from the nucleus and ablate their oncogenic function. We now show that such a modified peptide, cSNX1.3, can function as a therapeutic in an EGFR-dependent model of breast cancer and block RTK-induced migration and cell survival.

## Materials and methods

### Cell lines

MDA-MB-468 and H1975 cells were maintained in RPMI 1640 containing 10% FBS and 1% penicillin- streptomycin. BT20 cells were maintained in MEM containing 10% FBS and 1% penicillin-streptomycin. MCF10A were maintained in DMEM/F12 containing 5% donor horse serum 1% penicillin-streptomycin, 10 ng/ml cholera toxin (Sigma), 0.5 μg/ml hydrocortisone and 5.0 ng/ml EGF at 37 °C and 5% CO_2_. Chinese hamster ovary (CHO) and A549 cells were maintained in Ham’s F-12 media containing 10% FBS and 1% penicillin-streptomycin. All cell lines maintained at 37 °C and 5% CO_2_. To create the MDA-MB-468 EGFR knockdown line, lentiviral particles containing an IPTG-inducible shRNA against the 3’UTR of EGFR were purchased from Sigma (TRCN0000010329) (5’CCGGAGAATGTGGAATACCTAAGGCTCGAGCCTTAGGTATTCCACATTCTCTTTTTG-3’). MDA-MB-468 cells were incubated with hexadimethrine bromide (8 ug/mL) to increase transduction efficiency before viral particles were added at an MOI of 1. Transduced cells were then incubated with 1 mM IPTG for 2 days to establish knockdown and then remained in IPTG for the duration of the experiment. Cell lines were procured from ATCC. All cell lines are tested for mycoplasma every 6 months.

### Cell Viability

2 × 10^3^ cells were plated per well in a 96 well plate and left to adhere overnight. The following day, drug treatments were started and continued for 3 days. To measure the viability of the remaining cell population, 10% 3-(4,5-dimethylthiazol-2-yl)-2,5-diphenyltetrazolium (MTT) in media was added and incubated for 2 hours and conversion of MTT to formazan at 540 nm using a biotech Synergy LX plate reader.

### Subcellular Fractionation

Cells were trypsinized and washed with PBS, followed by centrifugation at 100 g (4 °C) for 5 minutes. The cell pellet was resuspended in ice-cold cytosolic fraction buffer (150 mM NaCl, 50 mM HEPES pH 7.4, 25 ug/mL digitonin, with Complete protease inhibitors (Roche) and phosphatase inhibitors (2.0 mM sodium orthovanadate, 10.0 μM ammonium molybdate and 10.0 mM sodium fluoride) and tumbled at 4 °C for 10 minutes, followed by centrifuged at 2000 g, 4 °C for 10 minutes and the cytosolic fraction transferred retained for analysis as the cytosolic fraction. The pellet was then washed twice in ice-cold PBS, resuspended in membrane fraction buffer (150 mM NaCl, 50 mM HEPES pH 7.4, 1% NP-40, with protease and phosphatase inhibitors) and incubated on ice for 30 minutes. This solution was centrifuged at 7000 g 4 °C for 10 min and the membrane fraction was retained for analysis as the membrane fraction. The pellet was washed twice with ice-cold PBS containing 150 mM NaCl and 1% NP-40, resuspended in nuclear fraction buffer (150 mM NaCl, 50 mM HEPES pH 7.4, 0.5% Sodium Deoxycholate, 0.1% SDS, and protease and phosphatase inhibitors) and sonicated for 15 seconds at and centrifuged at 13,000 g for 10 minutes at 4 °C and the supernatant was retained for analysis as the membrane fraction.

### Antibodies and reagents

Antibodies were purchased from the following sources: Cell signaling – EGFR-XP (human; D38B1), PARP (#9542), HSP90 (C45G5), Abcam – Histone H3 (ab1791), EGFR (mouse; Ab52894), Thermo –Bap31 (CC-1), βActin (A5441). Peptides were synthesized by Genscript and resuspended in sterile water at 1 mM for cell culture and 10 mg/mL in sterile saline for mouse injections. Lentiviral particles containing IPTG inducible shEGFR were purchased from sigma (Clone TRCN0000010329) (Target sequence: GAGAATGTGGAATACCTAAGG).

### Statistics

Statistics for the animal model was performed by the University of Arizona Cancer Center Biostatistics and Bioinformatics Shared Resources (BBSR). The cube root of the observed tumor burden was applied to normalize the raw values. The linear mixed-effects model was used to compare the tumor burden across time between cSNX1.3 treated mice and cPTD4 control mice. To determine whether the profile of the change across time differed between the cSNX1.3 and cPTD4 mice, the interaction of treatment and time was tested. For all cell culture experiments error bars represent the standard error of the mean across 3 experimental replicates.

### Migration assays

Prior to plating, a horizontal line was cut on the bottom surface of the plate across all wells with a scalpel as a guideline for imaging to ensure each time point covers a similar area. Cells were plated to confluency in a 24 well plate (~2.0 × 10^5^ cells) and allowed to adhere for 24 h and then serum-starved overnight. A vertical line of cells was removed with a p200 tip from each well by scoring through the cell layer. Each well was washed twice in PBS to remove lifted cells and cell debris, followed by imaging at 10x magnification (time 0). Treatments and ligands were then added, and cells were allowed to invade the wound area for 12 h at which time each well was imaged again. Images were analyzed in ImageJ to determine wound area at 0 and 12 h.

### SDS-PAGE and immunoblotting

Lysates were prepared as described, protein concentrations determined by BCA assay (Pierce) and resolved via standard SDS-PAGE [18]. Gels were transferred to either PVDF (Immobilon P or Immobilon FL) membrane and probed with the indicated antibodies and analyzed by either HRP-linked secondaries (Pierce) or IRDye secondaries (Li-Cor), followed by development in Dura Signal (Pierce) or IR (Licor).

### Protein expression and purification of human EGFR kinase domain

EGFR kinase domain protein (EGFRkin) was expressed in Sf9 cells using the Bac-to-Bac system (Invitrogen) as previously described [19]. Briefly pFastBacHT plasmid containing the cDNA of EGFR kinase domain with N-terminal His-tag was transformed into DH10Bac *E. coli* to generate bacmid DNA. P1 virus was generated by transfecting 2.0 mL of sf9 cells at 1.0 million cells/ml density with the purified bacmid DNA (200–3000 ng/μL). The P1 virus was harvested after 5–7 days following transfection. The P1 virus was diluted 100-fold into an sf9 cell culture at a density of 2.0 million cells/ml and cultured for 3 days. Supernatant containing the P2 virus was collected by centrifugation and diluted 40-fold into 1.0 L of sf9 cells at a density of 2.0 million cells/ml. After 2.5–3 days, cell pellets were harvested by centrifugation at 3500 x g at 4 °C for 20 min, and flash frozen in liquid nitrogen prior to storage at −80 °C.

To purify the EGFR kinase domain, Sf9 cell pellets (~10 g) was thawed on ice and homogenized with 2 passages on an EmulsiFlex-C5 (Avestin) in 50 mL of lysis buffer (50 mM Tris pH 7.5, 600 mM NaCl, 1 mM EDTA, 1 mM TCEP, protease inhibitors, DNAase, and 10% Glycerol). The lysate was centrifugated and supernatant was collected and loaded onto a 1.0 mL HisTrap FF column (GE) at a flow rate of 1.0 ml/min. Following loading, the column was washed with 20 column volumes of wash buffer (50 mM Tris pH 7.5, 600 mM NaCl, 20 mM Imidazole, 1 mM TCEP, and 10% Glycerol) and eluted with a linear gradient of elution buffers containing 20 and 300 mM imidazole. Fractions containing the EGFR kinase domain proteins were combined and concentrated to less than 1.0 mL. The concentrated proteins were loaded onto a Superdex 200 10/300 GL column (GE) equilibrated in buffer (20 mM Tris pH 7.5, 100 mM NaCl) for further purification. The purified proteins were concentrated, flash frozen in liquid nitrogen, and stored at −80 °C.

### Protein expression and purification of human SNX-BAR protein

The human SNX-BAR cDNA (residues 301–522) was synthesized (Genscript) and cloned into the pMAL-c5x vector encoding an N-terminal MBP tag followed by a TEV cleavage site. The MBP-SNX-BAR protein was expressed in *Escherichia coli* BL21(DE3) in Terrific Broth autoinduction media containing 1.0 g glucose, 2.5 g lactose, 2.0 mM MgSO_4_ and trace metal. Briefly, cells were cultured at 37 °C until OD~2.0 followed by low-temperature expression at 20 °C overnight and harvested for further purification.

Frozen cells were resuspended in lysis buffer (50 mM Tris-HCl pH 7.4, 200 mM NaCl, 1 mM EDTA, 1 mM TCEP, and protease inhibitor cocktail) and lysed in an Emulsiflex-C5 (Avestin). The supernatant was collected following centrifugation and loaded onto an Amylose column (5.0 mL). The loaded column was washed with 20 column volumes of wash buffer (50 mM Tris-HCl pH 7.4, 200 mM NaCl, 1 mM EDTA, 0.5 mM TCEP) followed by 2 column volumes of TEV digestion buffer (50 mM Tris-HCl (pH 7.4), 1 mM EDTA, 0.5 mM TCEP). 500 μg of TEV was incubated with the protein-bound amylose at room temperature overnight. The SNX-BAR protein was eluted from the amylose column with elution buffer (50 mM Tris pH 7.5, 200 mM NaCl, 10% glycerol and 1.0 mM TCEP). Uncut protein as well as MBP remained bound to the amylose and could be further eluted with 10 mM Maltose in the wash buffer. Purity of the proteins was confirmed by SDS-PAGE.

### Microscale thermophoresis (MST)

MST experiments were carried out using a Monolith NT.115 pico instrument (NanoTemper Technologies, Munich Germany). Nanotemper His-Tag Labeling Kit RED-tris-NTA 2^nd^ Generation dye was used to label His-EGFRkin. Briefly, 100 μL of His-EGFRkin (200 nM) was mixed with 100 μL of 40 nM Red dye and incubated for 30 min at room temperature. Standard-treated MST capillaries were used in the MST measurement. SNX Peptides or SNX-BAR protein were diluted in PBS-T buffer to make 16 1:1 serial dilutions from 20 μM. For each reaction, 10 μL of each diluted solution was mixed with 10 μL of the Red-dye labeled His-EGFRkin and loaded into standard-treated capillaries. Thermophoresis measurements were conducted at room temperature. *K*_d_ values were derived from the concentration-dependent changes in normalized fluorescence (F_norm_). Data were analyzed using NanoTemper MO. Affinity Analysis software.

### Bio-layer interferometry (BLI)

BLI experiments were carried out using an Octet RED384 instrument (ForteBio) with Octet NTA Biosensor. His-EGFRkin was prepared at 500 nM in binding buffer (20 mM Tris pH 7.5, 100 mM NaCl, 1.0 mM TCEP) and dispensed into a 96-well microplate (Griner). A second 96-well microplate contained cSNX1.3 peptide at 5 different concentrations of serial dilutions from 10 μM, glycine regeneration solution (pH 1.5), and binding buffer for baseline stabilization. Both plates were agitated at 1000 rpm during the entire experiment. Six Octet NTA (His-tag capture) sensor tips were used for the binding experiment. Sensor tips were first pre-hydrated in binding buffer for 2 min and then transferred to the His-EGFRkin-containing wells for loading (5 min). After a 3 min baseline wash in binding buffer, the binding signal was measured by dipping the His-EGFRkin-coated sensors into the wells containing the cSNX1.3 peptide at various concentrations. Binding was monitored over a 5 min association period, followed by a 10 min dissociation period, in which the sensors were dipped into wells containing only the binding buffer. *K*_d_ values were derived from fitting the binding kinetics curves using the Octet Data Analysis software.

### Animal studies

The transgenic mouse line WAP-TGFα (The Jackson Laboratory; Tg(WapTgfa)215Bri) were back crossed to C57BL/6 J background (N8). WAP-TGFα heterozygous males were bred with C57BL/6 J females to generate study animals and offspring were genotyped using primers against the WAP-TGFα transgene [18]. WAP-TGFα heterozygous females were housed continuously with C57Bl/6 J males to induce pregnancy, resulting in transgene expression. Mice were weighed and palpated 1x/week after their first pregnancy, until a tumor reached approximately 100 mm^3^ at which time they were entered into either the cPTD4 or cSNX1.3 arm of the study. To ensure randomized treatment arms we added mice to alternating treatment arms as they developed tumors, starting with the control cPTD4 arm. When a second mouse from the same litter developed a tumor, that animal was added to the opposite arm from its litter mate. The researchers were not blinded to the treatment arms. A total of 7 cPTD4 treated mice were entered with a total of 14 tumors, and 6 cSNX1.3 treated mice were entered with a total of 11 tumors. Once entered, mice were weighed, tumors measured, and injections given intravenously at 10 μg/g body weight 3x/week for 4 weeks. Mice were sacrificed when they reached a total tumor burden of 2000 mm^3^, when they had a tumor reach a diameter of 2 cm, or after four weeks of drug treatment. Upon sacrifice, all tumors were collected and fixed in 10% formalin; a portion of each tumor was homogenized in tissue lysis buffer to form tissue protein lysates. Tissues were paraffin-embedded and sectioned by the Tissue Acquisition and Cell Molecular Analysis Shared Resource at the University of Arizona Cancer Center. The University of Arizona IACUC committee performs oversite of animal experimental protocols with coordination from a veterinarian in compliance with AAALAC.

### 3D mammospheres

20,000 BT20 cells were suspended in mammosphere media (15 mL Mammocult media supplemented with 1.7 mL Proliferation supplement, 1.5 μL hydrocortisone, 30 μL heparin, and 150μL penicillin/streptomycin) and plated onto a low adhesion 6-well plate. After one week the number of spheres per well were counted. Cells from selected wells were then dissociated in trypsin EDTA and plated with fresh mammosphere media to develop secondary mammospheres.

## Results

### Sorting Nexin 1 BAR domain peptides inhibit cancer cell growth

**Fig. 1:**
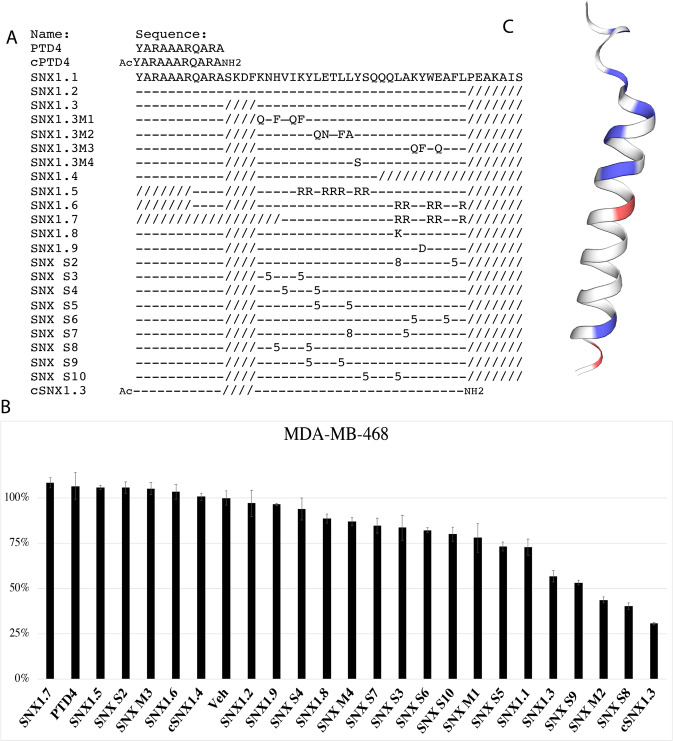
cSNX1.3 peptide inhibits cell viability in MDA-MB-468 cells. **A** Peptide sequence alignment of modified and stabilized peptides (−) indicates conserved residues, (/) indicates a deleted residue. Modified residues used for staples are (S)-2-(((9H-flouren-9-yl) methoxy) caronylamino)-2-methyl-hept-6-enoic acid [5] and (R)-2-(((9H-flouren-9-yl) methoxy) caronylamino)-2-methyl-dec-9-enoic acid [8]. PTD4 = Protein Transduction Domain. Ac = acetylation of 5’ end and NH2 = amidation of 3’ end. **B** MDA-MB-468 cells were treated with 10 μM of the indicated peptide for 3 days. Cell viability was measured using an MTT assay. Vehicle control represents 100%. **C**. A predicted peptide structure of SNX1.3 was generated using SWISSMODEL with negative (blue) and positive (red) residues highlighted.

Within the SNX1 BAR domain are dimerization domains as well as an EGFR binding domain [20]. To discover if the binding between EGFR and SNX1 was therapeutically targetable, we synthesized 3 SNX1-based peptides that overlapped the EGFR/SNX1 interaction domains (SNX1.1, 1.2 and 1.3). Each peptide was generated in tandem with a cell-penetrating peptide domain (PTD-4) to allow for intracellular uptake [16] (Fig. 1A, SNX1.1, 1.2 and 1.3). We next evaluated the ability of each peptide to reduce cell survival in the triple-negative breast cancer cell line MDA-MB-468 and BT20 (Fig. 1B and Supplementary Fig. 1A). Upon identifying that SNX1.3 inhibits the growth of BT20 cells, we began modifying the sequence to increase its efficacy and tested these modified peptides using the cell line MDA-MB-468, which has amplified EGFR [21] (Fig. 1B). To guide our modifications, we used the predicted peptide structure including negatively (blue) and positively (red) charged residues (Fig. 1C). We attempted to alter the sequence of the peptide surrounding the charged residues by charge replacement and sequence deletion but were unable to increase peptide efficacy. We then attempted to increase efficacy by either peptide stapling (substituting residues with non-natural amino acids followed by hydrocarbon linking) or end-capping with acetyl and amine groups. Of these, we found both C- and N-terminal peptide end-capping increased efficacy (Fig. 1B**)**, and further work was done with this peptide, cSNX1.3.

### cSNX1.3 induces tumor regression in WAP-TGFα transgenic mice

To evaluate the impact of cSNX1.3 on an immune intact mouse model of cancer, we utilized WAP-TGFα mice, a transgenic line whose mammary gland tumors are EGFR dependent [18]. Mice are continually bred to activate the pregnancy dependent WAP promoter, which drives expression of the EGFR ligand Transforming Growth Factor alpha (TGFα) strictly to the mouse mammary glands. This model stochastically forms unifocal mammary adenocarcinomas through a process that begins with mammary hyperplasia, followed by tumor formation over approximately 8 months. We established tumor-bearing females (as determined by forming a 100 mm^3^ tumor that does not regress upon subsequent palpation) and then treated them with either cPTD4 peptide as a control or cSNX1.3. First, we evaluated the potential toxicity of cSNX1.3 by treating C57Bl/6 J female mice with 5.0 or 10.0 μg/g body weight [3X/week, intravenous (IV) injections] with either cSNX1.3 or cPTD4 and weighing the animals for 3 days per week for 2 weeks (data not shown). No difference in weight, behavior, or grooming was observed, and we therefore used 10 μg/g body weight for subsequent studies.

**Fig. 2:**
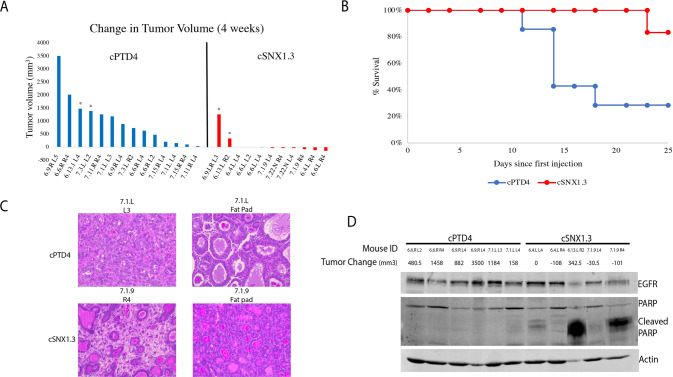
cSNX1.3 driven tumor regression in WAP-TGFα transgenic mice. Mice were bred continuously to induce transgene expression and palpated weekly for tumor formation. Once tumors reached 100 mm^3^, mice were entered into the study and given 10 µg/g body weight intravenous injections of either cPTD4 or cSNX1.3 3X/week. **A** Changes in tumor size from entry into study until end of study are shown for each individual tumor, (*) indicate tumors that entered the study at size greater than 500 mm^3^, all other tumors entered the study at ~100 mm^3^. **B** a Kaplan-Meier survival curve was generated showing when mice were sacrificed by either reaching tumor burden (2000 mm^3^) or the end of the study (*p* = 0.0002). **C** Upon sacrifice tumors were harvested and fixed in 10% formalin and embedded in paraffin. FFPE blocks were sectioned at 4 μM and stained with hematoxylin and eosin and imaged at 20x. **D** Protein lysates were generated from tumors upon the sacrifice and probed for the indicated proteins.

When tumors reached >100 mm^3^, IV injections began at 3X/week, 10 μg/g body weight and tumors were measured 3X/week with calipers. Animals were injected for 4 weeks or until they reached maximal tumor burden, defined as a total tumor volume >2000 mm^3^ or an individual tumor measuring >2 cm in diameter. In the cPTD4 treated mice, the tumors (*n* = 14) grew at an average of 30.8 mm^3^/day while in the cSNX1.3 treated mice tumors (*n* = 11) regressed at an average of 4 mm^3^/day (Supplementary Fig. 2B). Note that mice were weighed throughout the study and no impact on animal weight was observed in response to cSNX1.3 treatment (Supplemental Fig. 2A). The WAP-TGFα is a spontaneous model in which each tumor arises and progresses in a heterogeneous fashion. We therefore evaluated each tumor separately to determine the overall impact of cSNX1.3 treatment (Fig. 2A). We found that although cPTD4-treated tumors had a wide variety of tumor growth, all tumors grew by the end of the study. Conversely, 7/11 cSNX1.3-treated tumors regressed and 2/11 showed no change in tumor volume from the first to last measurement. Of note, 3 of the cSNX1.3 fully regressed as measured with calipers and confirmed during necropsy. Additionally, the 2 cSNX1.3 treated tumors that demonstrated growth through the study entered the study at greater than 500 mm^3^. In this study, mice were sacrificed after 4 weeks of injections or once they reached tumor burden, represented by the Kaplin-Meyer graph **(**Fig. 2B**)**. The increase in tumor burden was significantly different between cPTD4 and cSNX1.3 treated mice (*p* = 0.0002). While 5/7 cPTD4 mice reached tumor burden before the end of the study only 1/6 cSNX1.3 treated mouse reached the maximal tumor burden.

At the end of the study, mice were sacrificed, and tissues were collected and fixed in 10% buffered formalin or homogenized in tissue lysis buffer. Tissues were sectioned and evaluated for changes to tissue morphology in response to peptide treatment (Fig. 2C and Supplementary Fig. 3). All mice showed hyperplasia within fat pads that did not develop tumors as is normal with this model [22]. We found that tissue morphology was not significantly impacted by drug treatment, although some of the cSNX1.3 tumors had regressed fully to a hyperplastic mammary gland at the end of the study. No changes to tissue architecture were observed in the normal mammary gland between treatment arms, although, an increase in infiltrating leucocytes was observed in some tissues (data not shown). Analysis of protein expression in treated tumors found an increase in cleaved PARP, indicating cSNX1.3 was inducing apoptosis as a means of tumor regression (Fig. 2D). Representative tumors from the PARP-cleavage positive (cSNX1.3-treated) and negative (cPTD4-treated) are shown (Fig. 2C). Note that the tumor with the maximum level of PARP cleavage also showed the least reduction in size in response to treatment. Upon sacrifice, it was noted that this tumor mass was highly necrotic and acellular.

### cSNX1.3 competitively inhibits EGFR/Sorting Nexin 1 BAR domain interactions

**Fig. 3:**
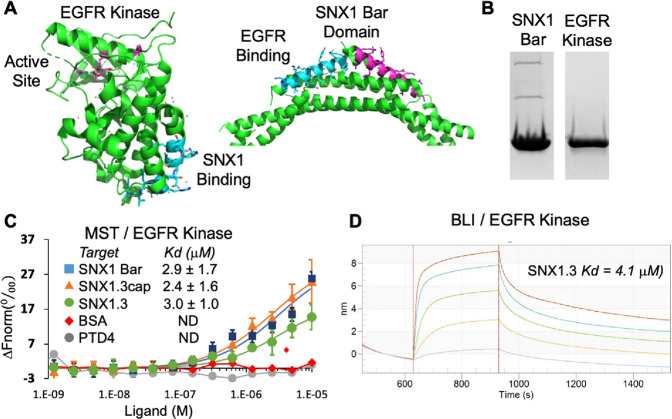
cSNX1.3 competitively inhibits binding of EGFR to SNX1. **A** Ribbon drawing of EGFR kinase domain (PDB ID 5CNO) and SNX1 Bar domain (PDB ID 4FZS). The proposed SNX1 binding site is well away from the kinase active site. **B** SDS page gels for purified SNX1 Bar domain (residues 301–522) and EGFR kinase domain (residues 672–998). **C** MST Binding curves of EGFR kinase domain (50 nM) with fluorescent dye attached through the His-tag titrated against the Bar domain, peptides SNX1.3 (capped), SNX1.3, and control PTD4, as well as control BSA. **D** Binding of SNX1.3 to the kinase domain was also measured by Bio-Layer Interferometry (Octet BLI), which yielded a similar dissociation constant to that measured by MST.

We next set out to confirm binding of cSNX1.3 to EGFR and cSNX1.3 inhibition of EGFR/Bar domain complex formation, following strategies similar to those employed for crystal structure determinations of the key domains (PDB ID 5CNO [19] and PDB ID 4FZS [23], Fig. 3A**)**. For this, we expressed and purified the kinase domain of EGFR using a baculovirus insect cell expression system (residues 672–998 with N-terminal His-tag followed by TEV cleavage site), and the SNX1 BAR domain using *E. coli* expression (residues 301–522 with N-terminal maltose binding domain followed by TEV cleavage site; Fig. 3B). The kinase domain was shown to be active using a coupled assay in the presence of membrane [24]. We first examined binding between EGFRkin and BAR domain, and EGFRkin and several peptides, using microscale thermophoresis (MST, Fig. 3C). As expected, binding between the purified domains was observed (*K*_d_ = 3 μM). Binding of cSNX1.3 to EGFRkin displayed similar affinity to that of the BAR domain (*K*_d_ = 2.4 μM). Bio-layer interferometry was also performed between the EGFR kinase domain and SNX1.3 which demonstrated similar binding (*K*_d_ = 4.1 μM) (Fig. 3D). Interestingly, in a competitive binding assay, we confirmed that 2.5 μM cSNX1.3 blocks SNX BAR binding to EGFRkin (data not shown). That sub-saturating concentrations of cSNX1.3 eliminates SNX BAR binding suggests a more complicated mechanism may be in play, such as slow release of cSNX1.3 from EGFRkin or interference with SNX BAR dimer formation.

### cSNX1.3 displays specificity for wildtype EGFR and cancer

**Fig. 4:**
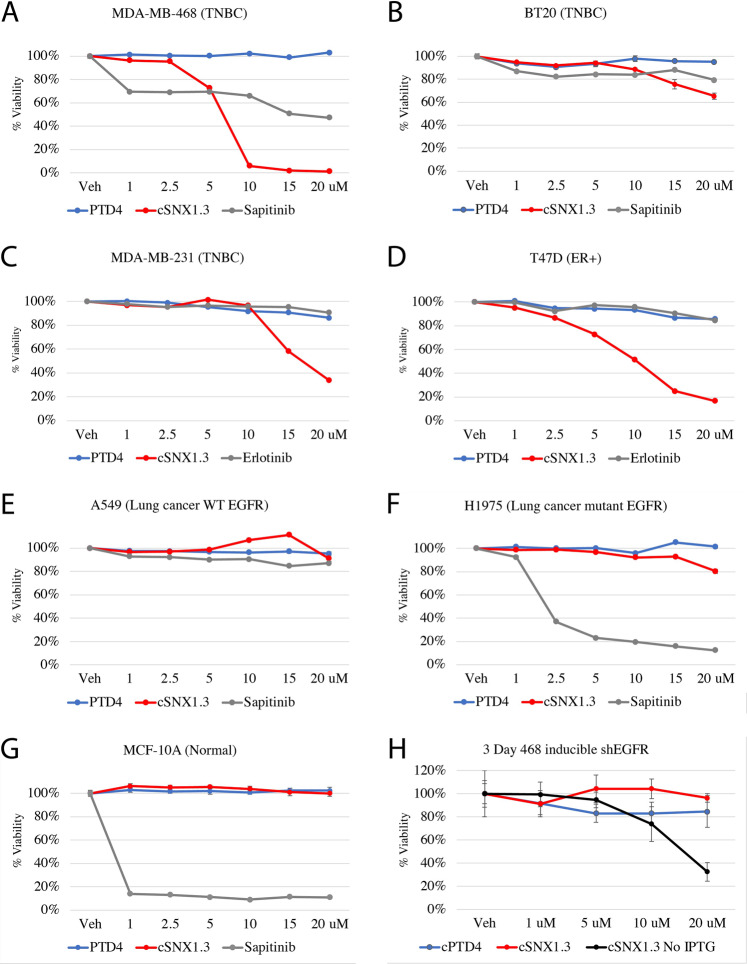
cSNX1.3 peptide inhibits EGFR driven cancer cell viability. **A–G** Cells were plated in a 96-well plate at 2000 cells per well. The indicated concentration of cPTD4, cSNX1.3, Sapitinib, or Erlotinib were added to the cells on day 0 and incubated for 3 days. Cell viability was then measured with an MTT assay, viability of treated cells were compared with the vehicle control. **H** MDA-MB-468 cells were plated and incubated+/− IPTG for 2 days prior to drug treatment to induce expression of an EGFR-targeted shRNA. Cells were then additionally treated with cPTD4 or cSNX1.3 for 3 days, and then viability was measured using an MTT assay.

We next evaluated the efficacy of cSNX1.3 compared to the tyrosine kinase inhibitor Sapitinib or Erlotinib (a Her-family and EGFR-specific TKI, respectively) in several cell lines. We utilized three triple-negative breast cancer cell lines MDA-MB-468, MDA-MB-231, and BT-20 (Fig. 4A–C). In addition to TNBC cell lines, we used the ER + breast cancer cell line T47D as the nuclear localization of EGFR is a mechanism of therapeutic resistance amongst ER + breast cancer (Fig. 4D). We then tested the lung carcinoma cell lines A549 (WT EGFR) and H1975 (T790M EGFR), the immortalized breast epithelial line MCF10A and parental CHO cells that lack EGFR expression with cSNX1.3, cPTD4 control, or Sapitinib (Fig. 4E–G and Supplementary Fig. 1B, C).

While both cSNX1.3 and Sapitinib inhibited cell survival, cSNX1.3 was more effective than Sapitinib in the breast cancer cell lines MDA-MB-468 (IC50 = 7.5μM) and BT20 (IC50 = 25μM). Interestingly, the breast cancer cell lines MDA-MB-231 and T47D (which have been previously characterized as resistant to the EGFR-specific TKI erlotinib) were sensitive to cSNX1.3. Importantly, cSNX1.3 had almost no impact on normal or immortalized cells (CHO and MCF10A), while Sapitinib was more effective at inhibiting cell growth in immortalized cells than in cancer cells. Additionally, while cSNX1.3 had minimal effect on the H1975 cell line, which has an EGFR driver mutation in the kinase domain (T790M), it was as ineffective as Sapitinib in the A549 lung cancer line with wildtype EGFR.

To evaluate the specificity of cSNX1.3 for EGFR, we next knocked down the expression of endogenous EGFR with an IPTG-inducible shRNA to the 3’UTR of EGFR in MDA-MB-468 cells (Supplemental Fig. 4). Cells with reduced EGFR displayed significantly less cell death in response to cSNX1.3 treatment (Fig. 4H). These data indicated that cSNX1.3 may be selective towards cancer expressing wildtype EGFR.

### cSNX1.3 inhibits trafficking of EGFR to the nucleus and mammosphere

**Fig. 5:**
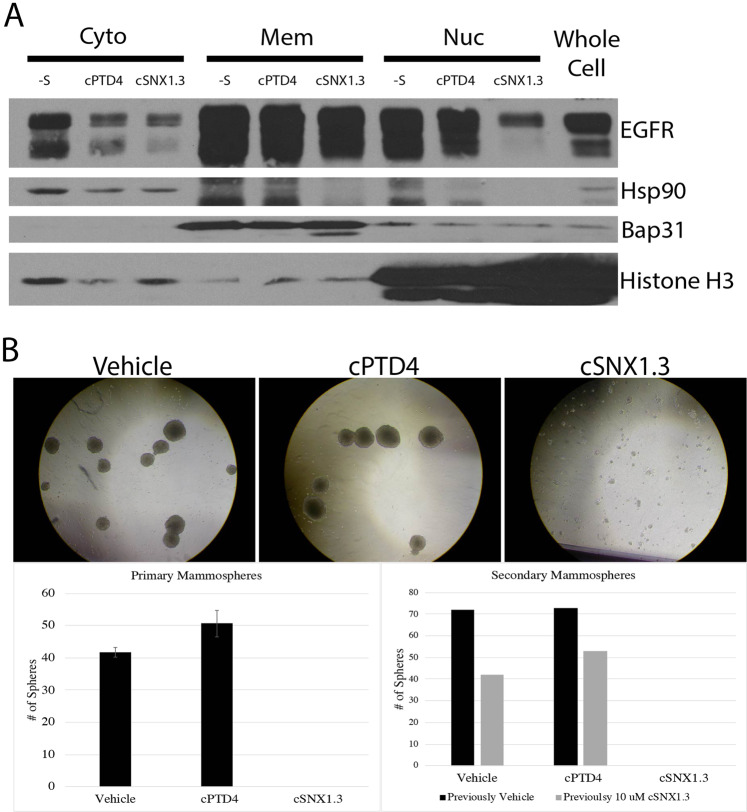
cSNX1.3 inhibits nuclear EGFR and survival signaling. **A** MDA-MB-468 cells were serum starved overnight then incubated with 20 ng/mL EGF and either cPTD4 or cSNX1.3 for 2 h. Cells were then fractionated and verified by HSP90 cytosolic protein, Bap31 membrane protein, or HDAC nuclear protein. **B** BT20 mammospheres were grown for 1 week in the presence of either vehicle, cPTD4, or cSNX1.3 and trypsinized, counted and replated. After the second week, the number of secondary mammospheres were counted.

We next investigated the capacity of cSNX1.3 to reduce EGFR nuclear localization. MDA-MB-468 were serum starved overnight then incubated with EGF and peptide for 2 h to allow for nuclear localization of EGFR. We then performed a subcellular protein fractionation to isolate cytosolic, membrane, and nuclear protein fractions (Fig. 5A). We observed a loss of nuclear-localized EGFR upon treatment with cSNX1.3, which was verified by Histone H3 versus Bap31 and Hsp90 for nuclear, membrane and cytosolic fractions, respectively. These data indicate that the peptide is impacting the species of EGFR that undergoes nuclear translocation.

To evaluate the effects of cSNX1.3 on cell survival, BT20 triple-negative breast cancer cells were evaluated by a mammosphere assay, which allowed us to evaluate cell survival in a non-adherent environment. While cells grew and formed mammospheres under the vehicle or cPTD4 treatment, no mammospheres were formed upon treatment with cSNX1.3 **(**Fig. 5B**)**. These spheres were then disassociated and re-plated to form secondary mammospheres. Interestingly although no spheres were formed in the presence of cSNX1.3 enough viable single cells were collected to plate three wells of secondary mammospheres. Cells that had been previously treated with the vehicle (water) or cSNX1.3 were plated for secondary mammospheres. Secondary mammospheres were treated with vehicle, cPTD4, and cSNX1.3 for an additional week. As expected, cells that were treated with cSNX1.3 on the second week showed no mammosphere formation. Interestingly, cells that had been treated with cSNX1.3 in the first week showed a reduction in sphere formation while treated with cPTD4 and vehicle in the second week, indicating some minimal lasting effect of cSNX1.3 on these cells.

### cSNX1.3 inhibits ligand-dependent migration of multiple RTKs

**Fig. 6:**
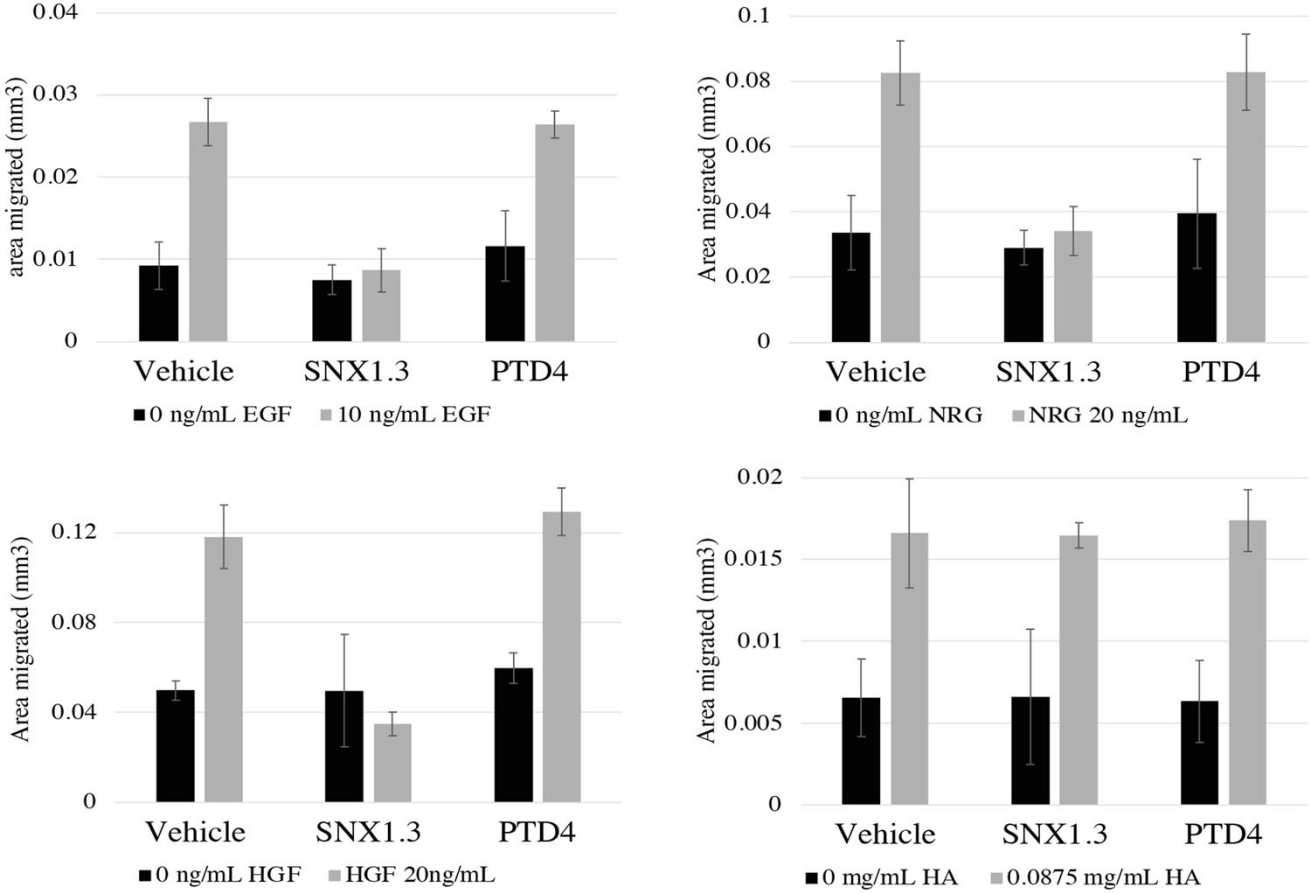
SNX1.3 inhibits RTK driven 2D cell migration. BT20 triple negative breast cancer cells were treated with either cSNX1.3, PTD4 control or vehicle (water) and either EGF-, Neuregulin-1 (NRG), Hepatic Growth Factor (HGF), or hyaluronic acid (HA)-induced migration on plastic was allowed for 12 h. Area migrated was measured with ImageJ.

EGFR activity is known to promote the activity of other RTKs, including those in the EGFR family (HER2, HER3 and HER4) and the c-Met receptor [25, 26]. The retrograde trafficking of EGFR has been previously described to promote cell migration in TNBC cells. Additionally, several RTK’s have been described to interact with SNX1. We therefore evaluated the ability of SNX1.3 to inhibit 2D migration, both in response to EGF as well as other migration-inducing ligands. Of note, sorting nexins have now been shown to directly regulate additional RTKs, including c-Met [14]. Cells were plated on plastic and allowed to migrate into an artificial wound over 12 h. Note that no impact on viability was observed via MTT in less than 24 hours, indicating any changes we observed were not due to viability (data not shown). We found that while EGF induced significant migration of BT20 cells in the presence of the control PTD4 peptide, no migration was observed in the presence of SNX1.3 peptides (Fig. 6). To determine if this inhibitory effect was restricted to EGFR-induced migration only, we also tested SNX1.3 for its ability to impact neuregulin (NRG1) and hepatic growth factor (HGF)-induced migration. NRG is the ligand for HER3 and HER4, and HGF the ligand for the c-Met receptor, all of which are receptor tyrosine kinases that can be directly activated by EGFR and are regulated by sorting nexins [14, 25, 26]. In addition, we tested soluble hyaluronic acid (HA)-induced migration, the ligand for CD44 [a non-kinase membrane receptor that induces migration [27]]. We found that while SNX1.3 inhibited migration from all three RTKs, it did not block CD44 migration. Note that all receptors undergo endocytosis, with the RTKs primarily undergoing clathrin-dependent endocytosis, while CD44 endocytosis is clathrin-independent [28].

## Discussion

In this study, we have discovered that a cell penetrating peptide designed to mimic the EGFR binding domain of SNX1 can induce tumor regression in vivo. This peptide also blocks cellular migration and survival in an EGFR- and tumor-specific manner. Treatment of tumor-bearing WAP-TGFα transgenic mice with cSNX1.3 induced tumor regression with no observable toxicity. Optimization of SNX1.3 through end-capping enhanced its use as a therapeutic and both SNX1.3 and cSNX1.3 were shown to directly bind the EGFR kinase domain. Mechanistically, cSNX1.3 treatment results in the loss of nuclear EGFR, which has been shown to activate several oncogenic pathways [29–35]. While evaluating the specificity of cSNX1.3 for EGFR, we found it also inhibits migration driven by other receptor tyrosine kinases that can function with EGFR, including HER3/4 and c-Met, but not the CD44 receptor. Together, these data indicate that targeting retrotranslocation of EGFR may be a potent therapeutic for EGFR-driven cancer.

We have previously demonstrated that blocking retrograde trafficking with Retro-2 reduced EGFR-driven cell migration and enhanced EGFR degradation in the lysosome [2]. The retrograde trafficking of EGFR towards the nucleus was a tumor-specific event that could be driven by the association with MUC1 or a loss of polarity, and these data prompted us to evaluate targeting this pathway therapeutically [18, 36, 37]. Other groups had previously demonstrated that SNX1 could interact with EGFR, promote its retrograde trafficking and regulate its trafficking to the lysosome [8, 9, 38]. The current work further supports the observation that retrograde trafficking of EGFR and other RTKs is an event that does not occur in normal polarized epithelium [37]. Importantly, this is in opposition to the impact of tyrosine kinase inhibitor, Sapitinib, which significantly reduces viability of normal cells.

Previous studies had found that blocking retrograde trafficking of EGFR significantly impacted EGF-dependent migration [2]. We therefore utilized migration assays to analyze the specificity of cSNX1.3 for EGFR versus other RTKs known to interact with EGFR and undergo retrograde trafficking. Of note, sorting nexins have now been shown to regulate additional RTKs, including c-Met [15, 38]. We found that while SNX1.3 inhibited migration from EGFR, HER3/4 and c-Met, it did not block CD44 migration. Note that all receptors undergo endocytosis, with the RTKs primarily undergoing clathrin-dependent endocytosis [39], while CD44 endocytosis is clathrin-independent [28]. As the SNX1 binding domain of EGFR is in the C-terminal region of its kinase, it is possible that RTKs with homology are being similarly targeted, but further work needs to be done to address this hypothesis.

In addition to migration, retrograde trafficking promotes cell survival (in MTT and colony forming assays) and thus may be of significant therapeutic value. We previously demonstrated that the WAP-TGFα transgenic mouse model is sensitive to knockout of the retrograde driver, Muc1, highlighting its use as a model of EGFR-dependent breast cancer [18]. We therefore utilized this model to test the in vivo efficacy of cSNX1.3 and found a significant reduction in tumor growth. Importantly we found several tumors completely regress upon cSNX1.3 treatment, indicating it may be an effective therapy for EGFR-driven cancers. The lack of observable toxicity in normal tissues, including the mammary glands where this pathway is also activated yet failed to induce tumors, reiterates the tumor specific effects of cSNX1.3.

Our data suggests targeting the retrograde trafficking of EGFR is a novel mechanism to target EGFR-driven cancers through the competitive inhibition of binding between the EGFR kinase and the SNX1 BAR domain. Homology of RTK kinase domains and inhibition of Her3/4 and c-Met driven migration suggest cSNX1.3 could be effective in the inhibition of other RTKs that work with EGFR to drive tumor progression.

## Supplementary information

Supplemental figure legends

Supplemental Fig 1

Supplemental Fig 2

Supplemental Fig 3

Supplemental fig 4

## Data Availability

Not Applicable
