# Supplementary material for: Sorting nexin-dependent therapeutic targeting of oncogenic epidermal growth factor receptor
Source: Cancer Gene Ther. 2022 Oct 17;30(2):267–76. doi: 10.1038/s41417-022-00541-7 (PMC9935382; doi:10.1038/s41417-022-00541-7)
Supplement: Supplementary file 1 — Supplemental figure legends [file 41417_2022_541_MOESM1_ESM.docx]

**Supplementary Data Figure legends**

**Supplementary Figure 1.** **A.** BT20 triple negative breast cancer cells were treated with SNX1.1, 1.2, or 1.3 for 3 days and their viability was measured via MTT analysis. **B.** CHO cells were treated with PTD4 or SNX1.3 for 3 days and their viability was measured via MTT analysis.

**Supplementary Figure 2.** **A.** WAP-TGFα were weighed 3x/week concurrent with tumor measurements and injections while in the study. **B.** Tumors were measured with calipers 3X/week. The average tumor growth rate for cPTD4 was 30 mm^3^/day and for cSNX1.3 was -4 mm^3^/day. Note that mice were sacrificed as tumor reached 2000mm^3^, asterisks indicate days that cPTD4 mice were sacrificed therefore reducing the number and size of tumors being averaged.

**Supplementary Figure 3.** Upon sacrifice, tissues were harvested and fixed in 10% formalin. Tissues were embedded, sectioned, and stained with H+E by the Tissue Acquisition and Cell Molecular Analysis Shared Resource (TACMASR) at the University of Arizona Cancer Center. H+E slides were then sent to the UC Davis pathology lab for pathological analysis.

**Supplementary Figure 4.** MDA-MB-468 cells were transduced with an IPTG inducible shRNA against the 3’ UTR of EGFR. These cells were incubated with 1mM IPTG for the indicated number of days and protein lysates were collected. Substantial knockdown was observed starting at 2 days and remained for 5 days.
