# Supplementary figures and images for: Sorting nexin-dependent therapeutic targeting of oncogenic epidermal growth factor receptor
Source: Cancer Gene Ther. 2022 Oct 17;30(2):267–76. doi: 10.1038/s41417-022-00541-7 (PMC9935382; doi:10.1038/s41417-022-00541-7)

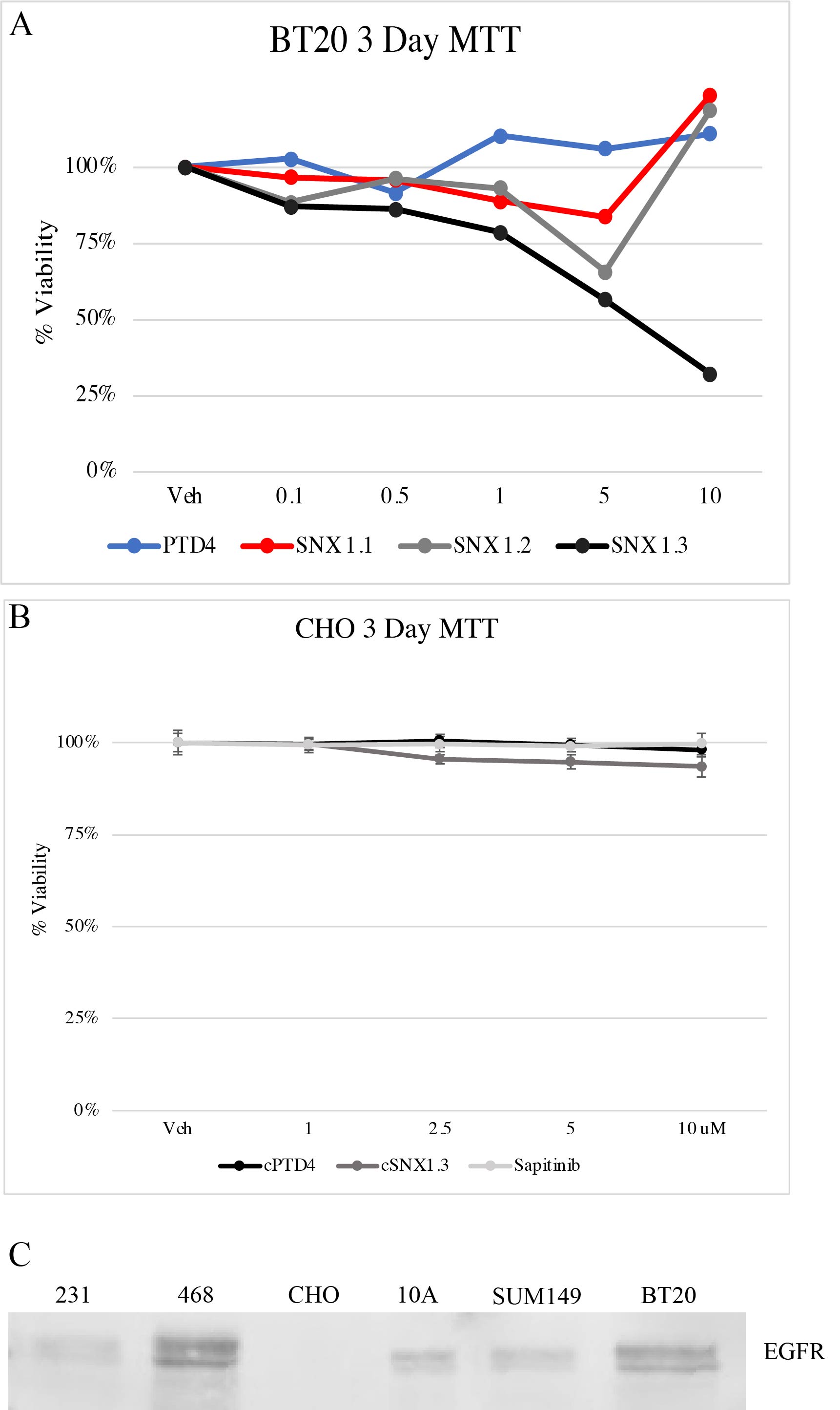

Supplement: Supplementary file 2 — Supplemental Fig 1 [file 41417_2022_541_MOESM2_ESM.jpg]

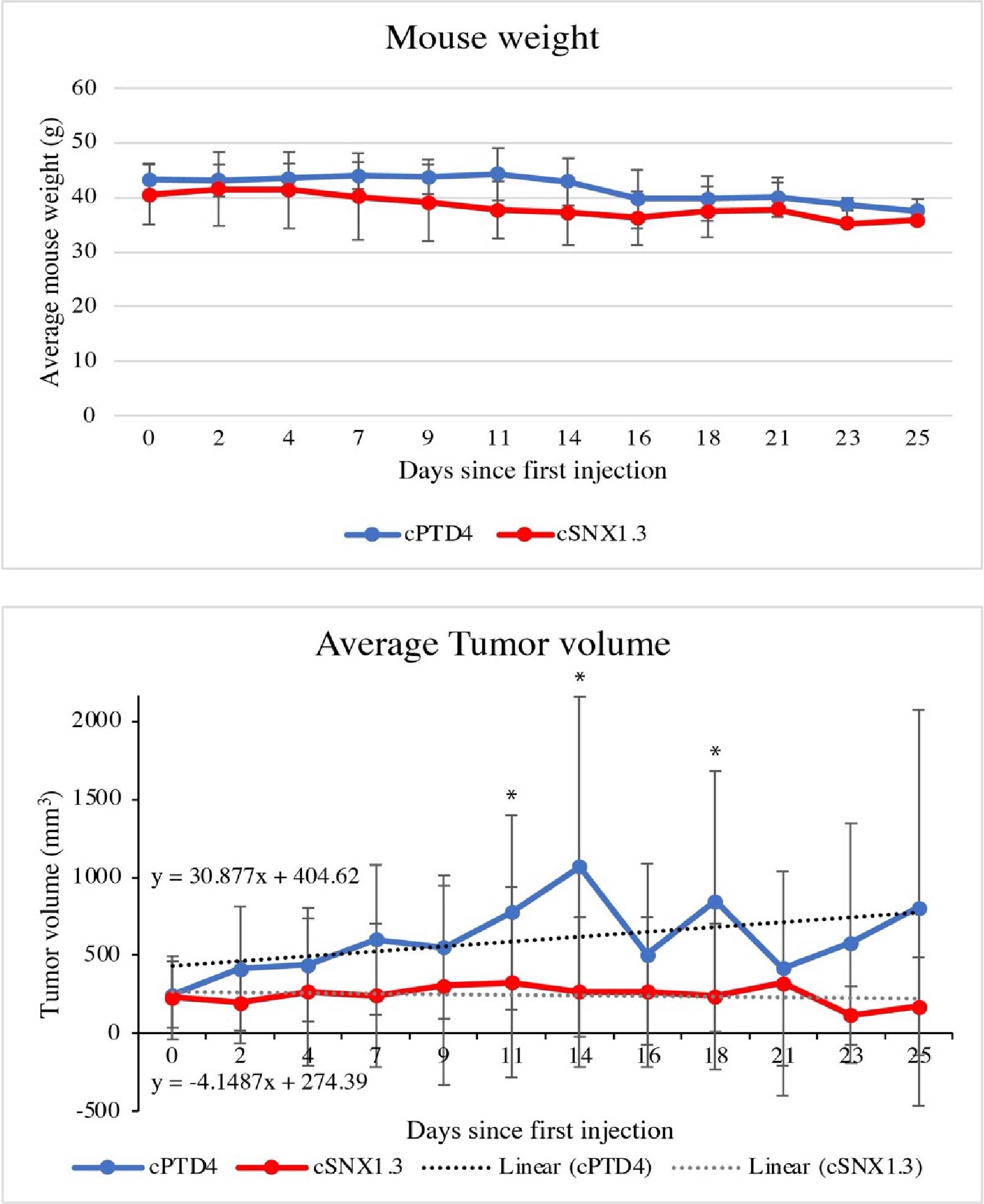

Supplement: Supplementary file 3 — Supplemental Fig 2 [file 41417_2022_541_MOESM3_ESM.jpg]

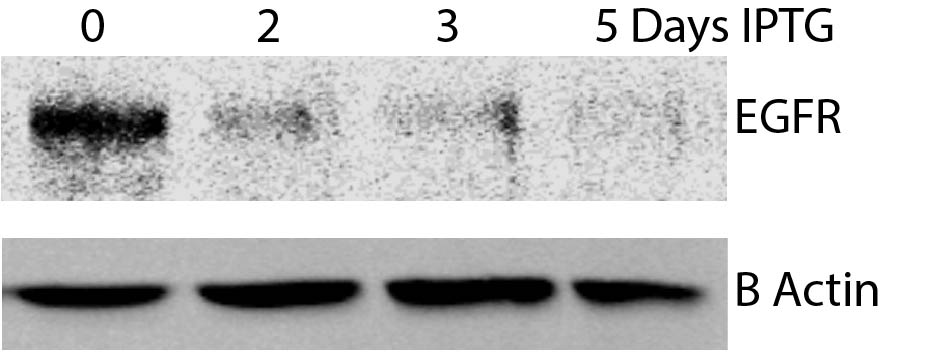

Supplement: Supplementary file 5 — Supplemental fig 4 [file 41417_2022_541_MOESM5_ESM.jpg]
